# Food and Constitution

**Published:** 1874-08

**Authors:** 


### FOOD AND CONSTITUTION.

If cattle are supplied with fodder, grass and water off granite gneiss or sandstone their bones are so brittle that very slight violence breaks them.

If pigs are fattened on flaxseed oil cake, the hams will taste of the oil. If any animal is fed on madder for a short time, and then omitted, there will be a reddish thread in the bones, answering to the time.

A man who has grown old in a limestone country has drank water enough, which, had it been evaporated, would have left his own weight in limestone.

A person eating bread made of the finest flour all his life will have brittle teeth, and the teeth of his children will be inferior; whereas, if bran bread only had been used, the teeth would have been strong, solid, enduring and beautiful, because of the ascertained fact that in five hundred pounds of fine flour there is only thirty pounds of bone making material. Five hundred pounds of bran contain one hundred and twenty-five pounds of material for making strong bones and solid teeth.

It is known that the natural color of silk can be changed by artificial means. If the silk worms are fed on grapevine leaves during the last twenty days of their spinning life, the silk will be of a beautiful red; or deep emerald green if fed on lettuce; if fed on the nettle, the silk is violet.

Although the body is but the casket of the mind, its condition does affect the mental and moral qualities to a greater or less extent. Hence, what a man eats and drinks has an influence on his moral character.

But the principle involved has a more far reaching tendency. The mental state of the mother affects the child she is nursing. A young mother became almost wild with excitement about some domestic occurrence. An explanation quieted her, and within half an hour she put her infant to her breast. In a few moments it was thrown into convulsions—never before or since.

There is in Boston a noble charity for the care of idiotic and deformed children, three fourths of whom are the children of parents one or both of whom were addicted to drunnkeness. Observation has elicited the fact, from different standpoints, that if a mother, while nursing, drinks beer or other forms of spirits "to make milk," the child, if it lives, has a feeble constitution, and in multitudes of cases "takes to drink."

If, then, a mother's food and drink, and state of mind during the nursing period, affect the bodily constitution as well as the mental and moral character, the facts given show that these same conditions of hers will have like effects for the mother before the nursing period, showing clearly the measure of parental responsibility for the physical, mental and moral character of their children—that parentage has its duties as well as pleasures.

